# Equal force generation potential of trabecular and compact wall ventricular cardiomyocytes

**DOI:** 10.1016/j.isci.2022.105393

**Published:** 2022-10-17

**Authors:** Jaeike W. Faber, Rob C.I. Wüst, Inge Dierx, Janneke A. Hummelink, Diederik W.D. Kuster, Edgar Nollet, Antoon F.M. Moorman, Damián Sánchez-Quintana, Allard C. van der Wal, Vincent M. Christoffels, Bjarke Jensen

**Affiliations:** 1 Department of Medical Biology, Amsterdam Cardiovascular Sciences, Amsterdam University Medical Centres, Amsterdam, the Netherlands; 2 Laboratory for Myology, Faculty of Behavioural and Movement Sciences, Amsterdam Movement Sciences, Vrije Universiteit Amsterdam, Amsterdam, the Netherlands; 3 Department of Physiology, Amsterdam Cardiovascular Sciences, Amsterdam University Medical Centres, Amsterdam, the Netherlands; 4 Department of Anatomy and Cell Biology, Universidad de Extremadura, Badajoz, Spain; 5 Department of Pathology, Amsterdam University Medical Centres, Amsterdam, the Netherlands

**Keywords:** Anatomy, Biology of human development, Developmental anatomy, Developmental biology, Mechanobiology, Medical imaging, Medicine

## Abstract

Trabecular myocardium makes up most of the ventricular wall of the human embryo. A process of compaction in the fetal period presumably changes ventricular wall morphology by converting ostensibly weaker trabecular myocardium into stronger compact myocardium. Using developmental series of embryonic and fetal humans, mice and chickens, we show ventricular morphogenesis is driven by differential rates of growth of trabecular and compact layers rather than a process of compaction. In mouse, fetal cardiomyocytes are relatively weak but adult cardiomyocytes from the trabecular and compact layer show an equally large force generating capacity. In fetal and adult humans, trabecular and compact myocardium are not different in abundance of immunohistochemically detected vascular, mitochondrial and sarcomeric proteins. Similar findings are made in human excessive trabeculation, a congenital malformation. In conclusion, trabecular and compact myocardium is equally equipped for force production and their proportions are determined by differential growth rates rather than by compaction.

## Highlights

• The trabecular and compact ventricular layers grow differentially during gestation
• Differential growth rates in the embryo result in temporary excessive trabeculation
• Normal and excessively trabeculated hearts show no difference in epitope expression
• Trabecular and compact cardiomyocytes can generate equal amounts of force

## Graphical abstract

**Figure:**
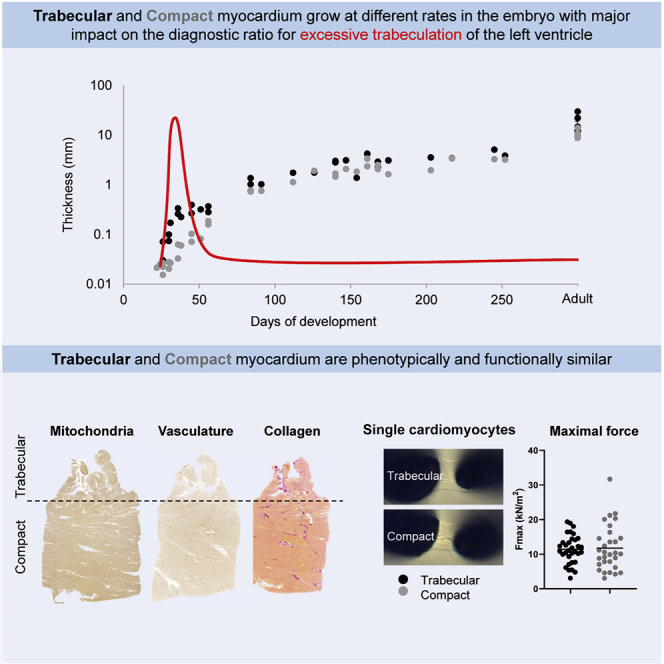

## Introduction

Vertebrate embryos always have a ventricular heart wall with a trabeculated surface on its luminal side (Icardo, 1996; Sedmera, 2011). In fully developed vertebrates, the proportions of the inner trabeculated layer to the outer compact layer exhibit strong evolutionary trends. Whereas the ventricular wall remains almost entirely trabeculated in cold-blooded vertebrates such as amphibians and most fish, it has independently become dominated by compact in the warm-blooded mammals and birds (Burggren et al., 2014; Sedmera et al., 2000). When primates are compared to other mammals, the trabecular layer is relatively thick and it may exceed the thickness of the compact layer (Anderson et al., 2017; Rowlatt, 1990; Shave et al., 2019). Approximately one-sixth of the left ventricular mass is trabecular in the normal human heart but this proportion can reach 40%, which is when the trabeculations are considered excessive (Grothoff et al., 2012; Riekerk et al., 2021).

How and when the proportions of trabecular and compact myocardium change during human gestation is largely unknown (Faber et al., 2021a). In the early embryo, there is an evolutionary conserved fast increase in trabecular myocardium (Sissman, 1970). Later, a process of compaction has been claimed to be important (Oechslin and Jenni, 2011). During compaction, the trabecular layer is said to diminish in thickness because a very substantial fraction of its myocardium becomes compact wall (MacGrogan et al., 2018; Sedmera et al., 2000; Towbin and Jefferies, 2017). A decrease in absolute thickness and volume of the trabecular layer, however, has not been measured (Faber et al., 2021a, 2021b). Should the alleged process of compaction fail, there is so-called noncompaction and the trabecular layer will persist in an excessive state at the cost of an abnormally thin compact wall (Chin et al., 1990; Towbin and Jefferies, 2017). However, experimental data do not support a critical role of trabeculations in the formation of compact wall (Anderson et al., 2017) and suggest instead that processes other than compaction are involved. For example, inhibited proliferation of the trabecular layer has much less impact on compact wall formation than inhibited proliferation of the compact layer (Tian et al., 2017). Also, an excessive trabecular layer does not preclude the formation of a normal compact wall (Choquet et al., 2018). Rather than a profound role of compaction, the experimental data is consistent with differences in growth rate of the trabecular and compact layer. Such differential growth rates, or allometry, are ubiquitous drivers of morphological change in evolution and development, including the fast ballooning of highly proliferative chambers from the embryonic heart tube (Gould, 1966; Moorman and Christoffels, 2003).

The trabeculated layer of the left ventricle receives much clinical attention because it potentially associates with poor pump function (Hussein et al., 2015; Kittleson et al., 2017). When the trabeculated layer thickness is expressed relative to the compact wall thickness in end diastole, the distribution of the general population exhibits a median ratio of approximately 1 with a long tail towards the excessively trabeculated (Anderson et al., 2017). The cases that make up the tail may be diagnosed as “noncompaction”, “hypertrabeculation”, or exhibiting “persistent sinusoids” even if these diagnoses are not fully overlapping and there is no consensus on the exact point along the tail at which the threshold for ‘excessive’ should be placed (D’Silva and Jensen, 2020; Finsterer et al., 2017). It has been proposed to further subdivide the criterion-positive population into groups based on pump function and the kind of associated mutations, *etc*., but there is no consensus on how it should be done (D’Silva and Jensen, 2020; Finsterer et al., 2017). Surprisingly, such subdivision of individuals encountered in the clinic has revealed that the largest fraction of these are so-called benign cases in which excessive trabeculation co-exists with normal pump function (Towbin et al., 2015). It is even more surprising that most individuals from the general population who are criteria-positive have normal pump function (D’Silva et al., 2020; de la Chica et al., 2020; Weir-McCall et al., 2016; Woodbridge et al., 2019) and the presence of excessive trabeculation in symptomatic patients does not provide additional prognostic value above that of the presence of left ventricular fibrosis and dilatation (Arbustini et al., 2016; Aung et al., 2020; Grigoratos et al., 2019). The alleged association to poor pump function appears to presume the trabecular myocardium is weaker than the compact myocardium. Only the relative (but not absolute!) thinness of the compact wall, for example, is emphasized in most diagnostic criteria of excessive trabeculation (Chin et al., 1990; Finsterer et al., 2017; Jenni et al., 2001; Oechslin and Jenni, 2011; Petersen et al., 2005). In addition, greater strength of the compact myocardium has been presumed to underlie the developmental change towards a greater proportion of compact myocardium (Wessels and Sedmera, 2003). It is mostly a hypothetical notion, however, that the trabecular myocardium should be relatively weak. If, in contrast, there is an equal force generation capability of trabecular and compact wall ventricular cardiomyocytes, this might explain why most hearts with excessive trabeculation exhibit normal pump function.

Our aim was to investigate left ventricular free wall development during gestation to measure the magnitude and timing of compaction. Because the diagnosis of excessive trabeculation depends on ratio in layer thickness (Jenni et al., 2001; Petersen et al., 2005) and calculated ventricular volume as proxy for myocardial mass (Jacquier et al., 2010; Grothoff et al., 2012), those were also investigated. We compared the findings from human to the growth curves we established for mouse and chicken, which are the most-used animal models to study cardiac development which have compact ventricular walls. Finally, we investigated if there are functional differences between trabecular and compact myocardium.

## Results

### Ventricular wall growth

**Figure 1:**
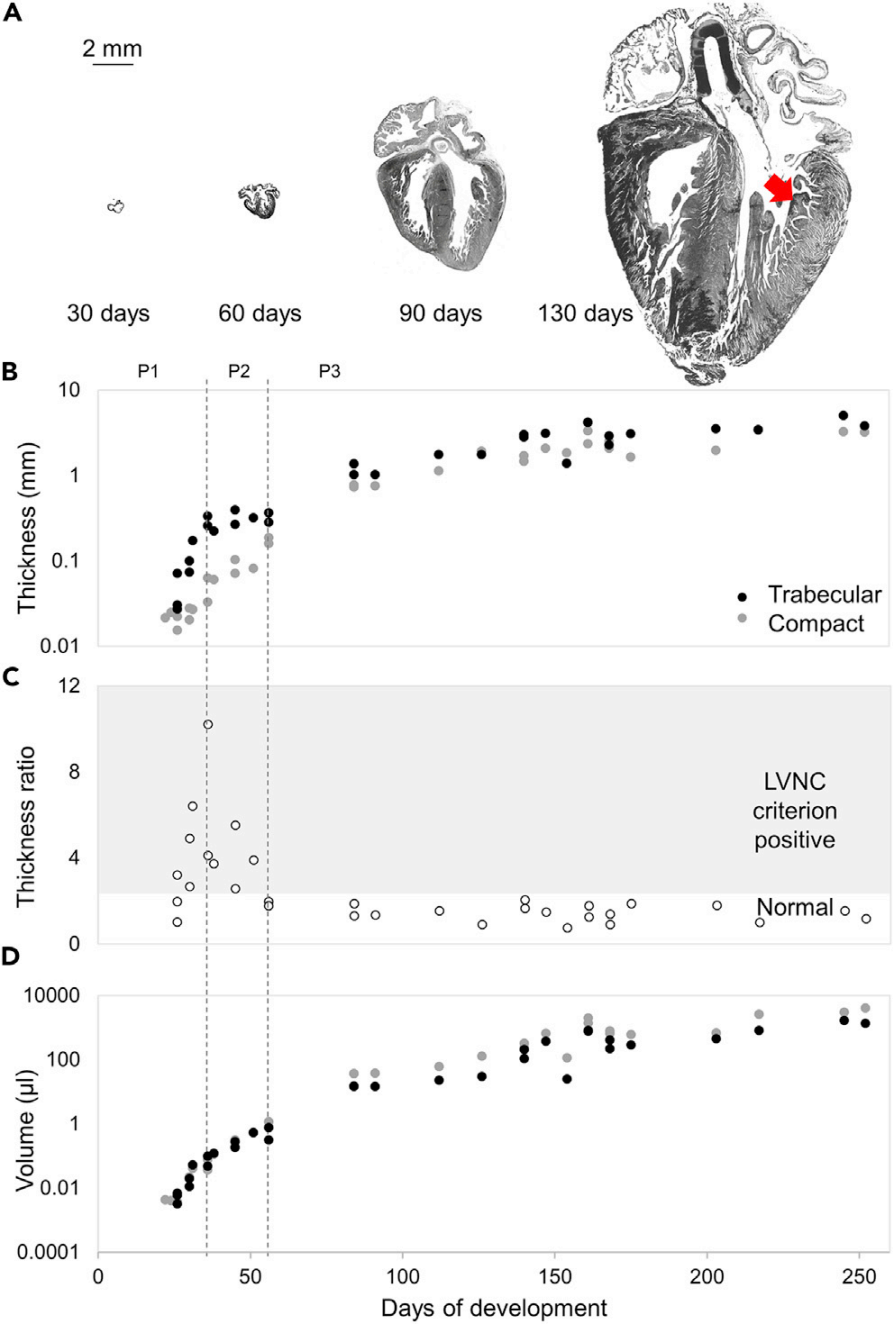
Gestational growth of the human heart (A) Human gestational hearts displayed on the same scale (scale bar 2 mm), showing continuous growth of the heart, including the trabeculated layer (a single trabeculation is indicated with the red arrow). (B and C) In early chamber development, between 22 and 31 gestational days (P1), the trabecular layer thickness increases more than the compact (B) which is reflected in a thickness ratio (C) that exceeds the threshold for excessive trabeculation (Petersen et al., 2005). In late embryogenesis, between 32 and 56 gestational days (P2), the trabecular layer thickness plateaus (B) whereas the compact layer continues to thicken, this causes a drop in thickness ratio to below excessive trabeculation. In the foetus (P3), the trabecular and compact layer thickness increase similarly (B) which stabilizes the thickness ratio (C). (D) Calculated trabecular and compact volumes continuously increase in gestation, even in P2 when the thickness ratio drops substantially.

The human heart grows from 0.07 to 4.7 cm in length between 20 days and 36 weeks of gestation. Early embryonic hearts fit *in toto* into individual trabeculations of the late fetal heart (Figure 1A). The trabecular layer thickness never decreases as would be expected if compaction took place (Figure 1B). Very substantial changes to the excessive trabeculation diagnostic ratio, however, did occur in the embryonic period (Figure 1C). This was because of changes in layer growth rates. From approximately 22–31 gestational days, the trabecular layer increased faster in thickness than the compact layer (16 ± 9 μm/day, F = 21.26, 1 degree of freedom versus 0 ± 1 μm/day, difference p < 0.001, F = 0.64, 1 degree of freedom). This caused the thickness ratio to increase to 10.2. This value is much higher than the clinical cut-off value of 2.3 used to indicate excessive trabeculation (Petersen et al., 2005). From approximately 32 to 56 gestational days, the growth rates of both layers were not significantly different (trabecular myocardium 2 ± 7 μm/day, F = 0.87, 1 degree of freedom, compact myocardium 6 ± 3 μm/day, F = 0.79, 1 degree of freedom, difference p = 0.309). However, the trabecular thickness growth rate did not correlate with age (p = 0.387), while the compact thickness was positively correlated to age (p = 0.003). This indicates that the trabecular thickness remained constant, causing the thickness ratio to decrease to below the threshold for excessive trabeculation (Figure 1C). Since the ventricles continuously expand, the compact and trabecular layer volumes have to grow to maintain a stable layer thickness, which is seen most clearly in late embryonic development where the trabecular layer thickness is stable and the trabecular volume grows (Figures 1D, Online S2). In the fetal period, both the trabecular and compact layer grew continuously with no difference in the rate of thickening (trabecular slope 19 ± 8 μm/day, F = 29.84, 1 degree of freedom, compact slope 15 ± 5 μm/day, F = 44.11, 1 degree of freedom, difference p = 0.327). This stabilizes the thickness ratio below the threshold for excessive trabeculation.

**Figure 2:**
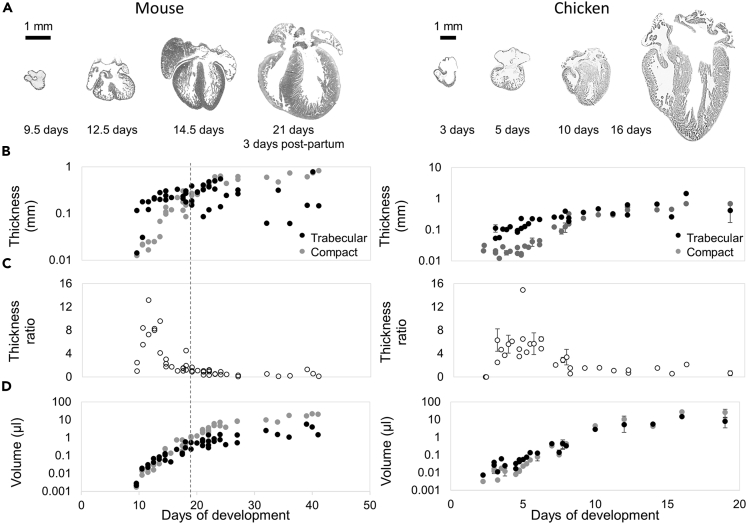
Growth of the mouse and chicken heart (A) Absolute size increase of mouse hearts between 9.5 days of gestation and 23 days post natally (all images are on the same scale, scale bar 1 mm), and chicken hearts between 2 and 19 days of gestation (all images are on the same scale, scale bar 1 mm). (B) In both the mouse and chicken embryo the trabecular thickness rapidly increases. Towards the end of fetal development, even continuing till after birth in mouse (dotted vertical line), the trabecular thickness stabilizes. The compact wall thickness continuously grows. (C) In the embryo, the maximum peak ratio of median trabecular layer thickness over median compact layer thickness is reached whereas in the fetus the ratio lowers and stabilizes. (D) Calculated trabecular and compact volumes continuously increase.

In mouse, the most used animal model for studying excessive trabeculation, and chicken, the animal model in which the process of compaction of the base of the trabeculations was demonstrated for the right ventricle (Rychterová, 1971), the growth curves of the left ventricular trabeculated and compact myocardium had similar trajectories to human. This was also illustrated by the transient peak in layer thickness ratio in the embryo (Figure 2). No decrease in absolute trabecular layer thickness was seen (Figure 2B), even if the trabecular layer was labeled to be excessive in younger stages and exceedingly regressed in older stages (Online Figure S3). Although the post-natal mouse hearts appeared to show a larger variation in trabecular layer thickness between specimens than fetal human and late-gestational chicken, at no point was there a re-increase in thickness ratio (Figure 2C) or a decrease in trabecular volume (Figure 2D).

### Trabecular and compact myocardial function related phenotype

**Figure 3:**
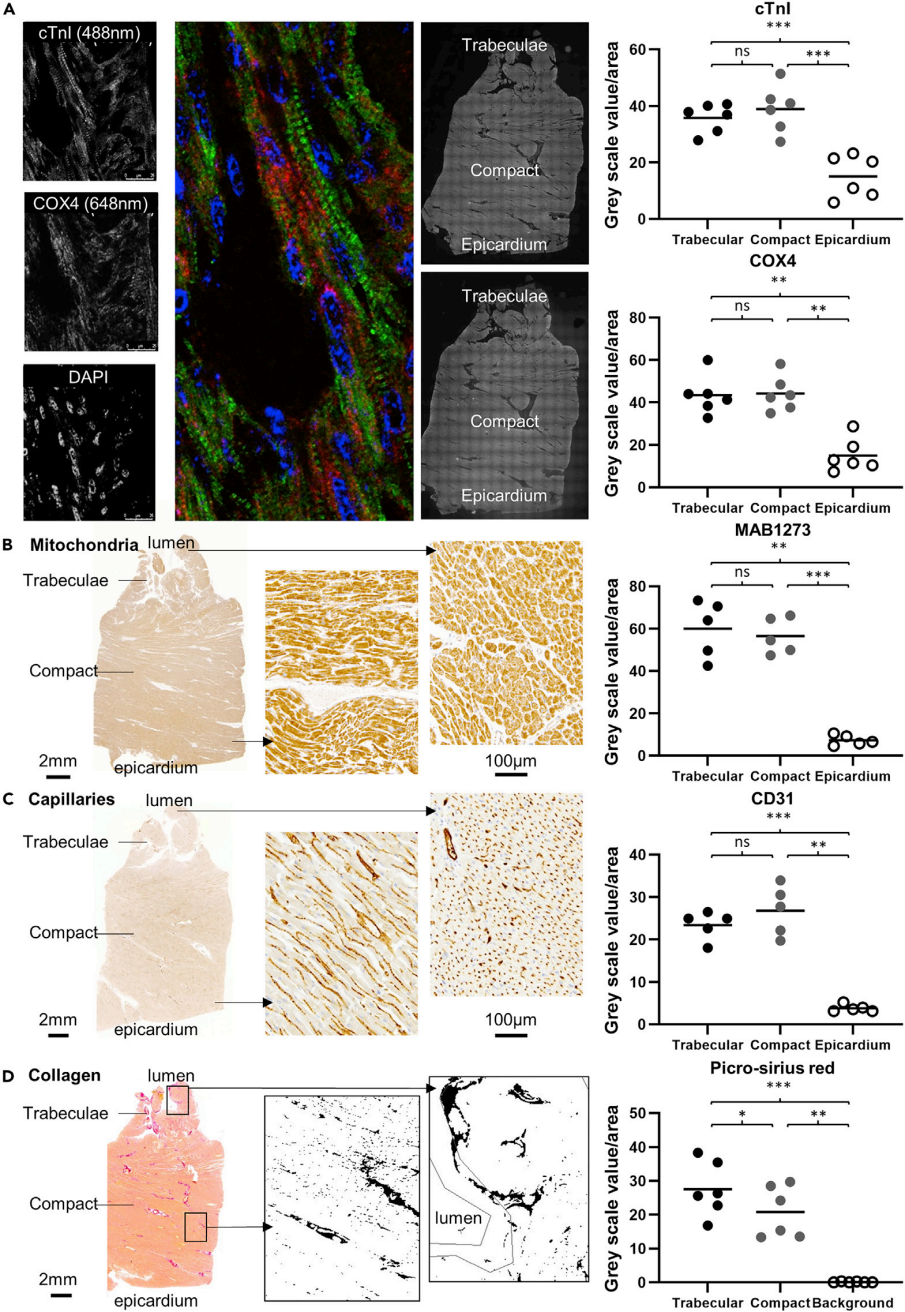
Trabeculated and compact tissue is similar in adult human (A–C) Staining intensity of sarcomeric protein cTnI (N = 6), mitochondrial protein COX4 (N = 6) (A), mitochondrial surface protein MAB1273 (N = 5) (B) and endothelial marker CD31 (N = 5) (C) are similar in compact and trabecular myocardium (p = 0.247, p = 0.857, p = 0.802 and p = 0.283 respectively) and greater than in epicardium. (D) Picrosirius red stains (N = 6) showed relatively more collagen in the trabecular wall compared to the compact wall (p = 0.038), likely because of insertion of tendinous cords in the trabeculations. All shown sections are from the same tissue block and shown on the same scale (scale bar 2 mm). In the confocal images of A, the scale bars are 25 μm. Statistical analysis: One-way ANOVA, Tukey’s corrected. Horizontal lines represent means, ∗ = p ≤ 0.05, ∗∗ = p ≤ 0.01, ∗∗∗ = p ≤ 0.001.

To test for similarity in myocardial phenotype, we assessed the relative abundance of proteins related to ATP production (mitochondrial protein), force generation (sarcomeric protein) and blood supply (vascular endothelium) in adult human trabecular and compact myocardium. Since sarcomeric proteins occur in tissues with a fixed stoichiometry (Palermo et al., 1996), values on cardiac troponin I (cTnI) are a good indicator of total sarcomeric protein in a cell. The staining signal was not different between trabecular and compact myocardium (p = 0.247, F = 62.24, 2 degrees of freedom), but greater than that of non-myocardial tissue (Figure 3A). Similarly, there was no difference in the signal intensity of mitochondrial protein COX4 (p = 0.857, F = 36.89, 2 degrees of freedom) (Figure 3A), mitochondrial surface protein MAB1273 (p = 0.802, F = 63.56, 2 degrees of freedom) (Figure 3B), and endothelial protein CD31 (p = 0.283, F = 86.67, 2 degrees of freedom) (Figure 3C). The distance between capillaries was also similar, indicating similar cardiomyocyte size and similar microvascular density (17.9 ± 1.3 μm in trabecular versus 18.9 ± 2.9 μm in compact myocardium, p = 0.284, T value = 1.237, 4 degrees of freedom, N = 5, two-tailed paired Student’s T-test). Only collagen was slightly more abundant in the trabecular layer, which, however, contained a few collagenous tendinous cords (p = 0.038, F = 50.05, 2 degrees of freedom, Figure 3D).

### Trabecular and compact myocardial function

**Figure 4:**
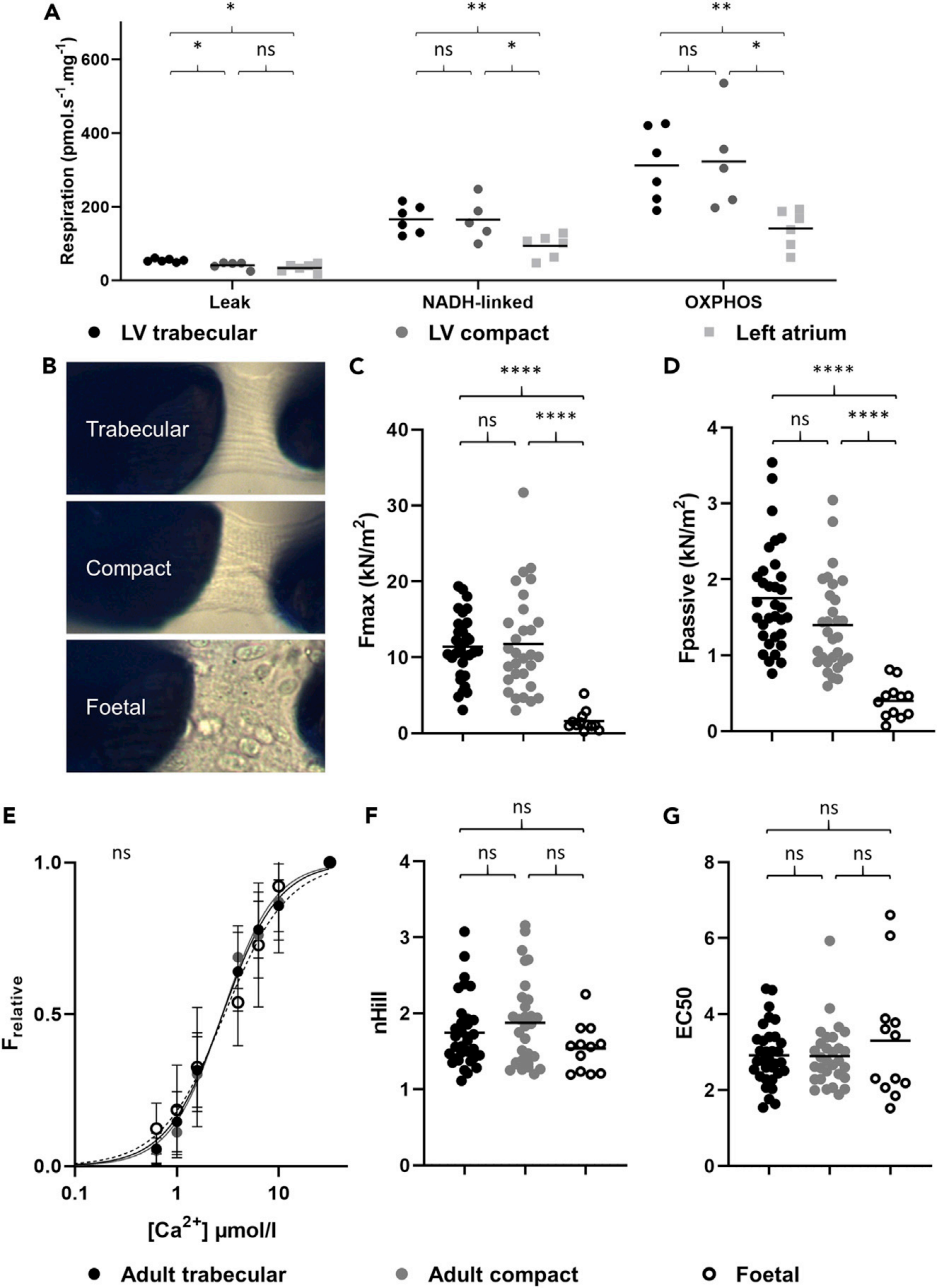
Functional analyses in mouse (A) Mitochondrial activity assay showed only a significant difference between trabecular (N = 6) and compact myocardium (N = 5) in leak respiration (p = 0.033), for NADH-linked respiration (p = 0.999) and maximal oxidative phosphorylation (OXPHOS, p = 0.989) there were no significant differences. Trabecular myocardium always had higher activity than left atrial myocardium (N = 6) (p = 0.027, p = 0.008, p = 0.008 for each treatment respectively) while compact myocardium was only more active after leak respiration (p = 0.453, p = 0.026, p = 0.032 for each treatment respectively). Statistical analysis per condition: Mixed-effects analysis, Tukey’s corrected. (B) Single adult trabecular and compact cardiomyocytes and a cluster of fetal cardiomyocytes as glued between the measuring needles (the actual height of each image is approximately 20μm). (C) The F_max_ of adult trabecular (N = 31, 6 specimens) and compact cardiomyocytes (N = 30, 6 specimens) is similar (p = 0.959) while the fetal cardiomyocytes are significantly weaker (N = 12, 2 specimens, p < 0.0001). (D) The F_pas_ of adult trabecular and compact myocardium is not different (p = 0.060) whereas fetal cardiomyocytes are significantly more compliant (p < 0.0001). (E) Calcium dose response curves of all groups were not different (p = 0.075). (F) The Hill coefficients (nHill) of all groups were not different (p = 0.136) (F). The calcium EC50 of all groups were not different (p = 0.430). Statistical analysis: One-way ANOVA, Tukey’s corrected. Horizontal lines represent means, ∗= p ≤ 0.05, ∗∗= p ≤ 0.01, ∗∗∗= p ≤ 0.001.

We next tested the hypothesis that the trabecular and compact myocardium have similar mitochondrial and contractile function. Myocardial work requires ATP and is strongly correlated with cellular oxygen consumption (Bayeva et al., 2013). Therefore, we compared mitochondrial respiration in mouse trabecular, compact and atrial myocardium. Maximal NADH-linked respiration, oxidative phosphorylation capacity and uncoupled respiration did not differ between trabecular and compact myocardium (p = 0.999, p = 0.989, p = 0.997 respectively), while mitochondrial respiration in the left atrial wall was significantly lower (Figure 4A). Leak respiration was higher in trabecular than in compact myocardium (p = 0.033) and the left atrial wall (p = 0.027).

To assess the force generating capacity of trabecular and compact myocardium, we compared force production of isolated cardiomyocytes. There was no difference between adult compact and trabecular cardiomyocytes in maximum force production (F_max_, p = 0.959), average passive force, (F_pas_, p = 0.060), or calcium sensitivity (p = 0.075) (Figures 4B–4G). Trabecular myocardium is thought to be more embryonic-like than compact myocardium (Oechslin and Jenni, 2011), but both trabecular and compact adult cardiomyocytes had much greater maximum force production than fetal ventricular myocardium (p < 0.001 and p < 0.001, F = 19.83, 2 degrees of freedom) (Figures 4C and 4D).

### Trabecular and compact myocardium are not different in human excessive trabeculation

**Figure 5:**
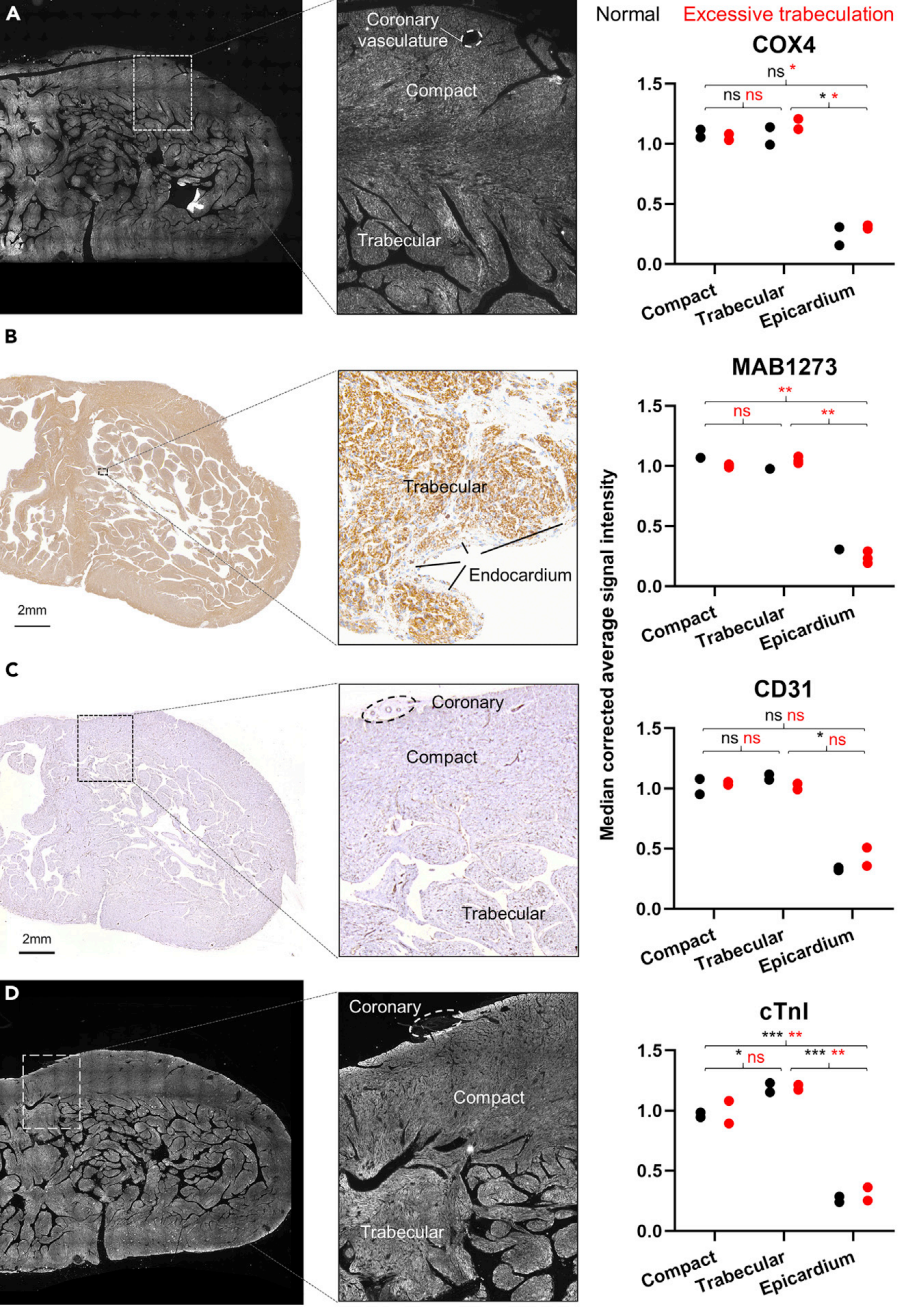
Trabecular and compact myocardium are not different in human fetusses with excessive trabeculation (A–D) Staining intensity differences between compact and trabecular myocardium in normal hearts (black) and hearts with excessive trabeculation (red) of mitochondrial protein COX4 (respectively p = 0.978, F = 40.53, 2 degrees of freedom and p = 0.156, F = 1050, 2 degrees of freedom) (A), mitochondrial surface protein MAB1273 (respectively no test, N = 1 and p = 0.133, F = 397.2, 2 degrees of freedom) (B), endothelial protein CD31 (respectively p = 0.719, F = 80.25, 2 degrees of freedom and p = 0.796, F = 60.92, 2 degrees of freedom) (C) and sarcomeric protein cTnI (respectively p = 0.023, F = 292.7, 2 degrees of freedom and p = 0.203, F = 52.40, 2 degrees of freedom) (D). All shown sections are from the same tissue block and shown on the same scale (scale bar 2 mm). Statistical analysis: One-way ANOVA, Tukey’s corrected. All categories, except MAB1273, were N = 2. For MAB1273 normal was N = 1 and excessive trabeculation was N = 3. ∗= p ≤ 0.05, ∗∗= p ≤ 0.01, ∗∗∗= p ≤ 0.001.

Because trabecular and compact myocardial function were not different in normal hearts, we hypothesised the two layers would not be different in human cases of excessive trabeculation either. We found no significant differences between compact and trabecular myocardium regarding the COX4 (Figure 5A), MAB1273 (Figure 5B), and CD31 (Figure 5C) signal intensity in normal and excessively trabeculated cases. There was only a significantly higher staining intensity for cTnI in normal trabecular myocardium compared to normal compact myocardium (p = 0.023, F = 292.7, 2 degrees of freedom, Figure 5D).

## Discussion

Here, we show for the first time that in the fetal period and postnatally the left ventricular trabecular and compact myocardium are surprisingly similar in terms of growth and key aspects of their functional phenotype. While this is surprising when premised that a greater extent of trabeculation has a negative impact on pump function, our findings are fully consistent with the growing number of cohort studies showing no or only very slight correlations between extent of trabeculations and pump function (Chuang et al., 2012; de la Chica et al., 2020; Zemrak et al., 2014). Nonetheless, excessive trabeculation and non-ischemic cardiomyopathy clearly can co-exist. We would suggest that in such setting the myopathy likely affects all myocardium. That is, we consider it unlikely that the cause of disease is a trabeculopathy. Although ventricular dilatation and fibrosis have prognostic value in predicting adverse outcomes, the extent of trabeculation in itself does not (Aung et al., 2020; Grigoratos et al., 2019). Excessive states of trabeculation are thought to result from failed compaction of embryonic trabeculations. However, we could not measure compaction while we do show that differences in growth rate have a tremendous impact on the diagnostic wall ratio.

### Differential growth rate determines left ventricular wall development

Histological techniques showed continuous growth of both trabecular and compact myocardium during development in human, mouse, and chicken. This indicates that differential growth shapes the left ventricular wall. Differential growth also forms other chambers of the heart and, more generally, embryonic organs, limbs, head and more (deBakker et al., 2016; Christoffels et al., 2000). We found no evidence for compaction or a loss of myocardium of the trabecular layer, as would be expected when compaction would occur, though we did see very substantial changes to the wall thickness ratio in the embryo. So, differential growth can lead to excessive trabeculation in the absence of compaction.

Since there may be distinct phases in trabecular layer thickness growth, natural and pathological variation in the duration of these phases may impact on the wall thickness ratio. If, for example, there is an unusually short duration of the period we call P2, which sees a substantial drop in the wall thickness ratio, the prediction would be an excessive ratio. Because the ratio does not indicate which layer behaves abnormally, patients with excessive trabeculation may have either excessive trabeculation, an underdeveloped compact wall, or both (Hu et al., 2017; Luxán et al., 2013; Rhee et al., 2018). Also, a longer duration of fast trabecular growth does not necessarily imply inferiority of the trabecular myocardium. In fact, in our cases of excessive trabeculation, the trabecular and compact layers appeared equally equipped with vasculature, mitochondria and sarcomeric protein. Because of the perfusion from the coronary circulation, the individual fetal and postnatal trabeculations can be much thicker than the avascular embryonic trabeculations (Goo et al., 2009). Also, they lose some of the protein expression that sets them apart from the compact wall in the embryo, such as ANF and CX40, which becomes restricted to the Purkinje system and is thus absent from the other kinds of trabecular muscle, namely the trabeculae carneae and papillary muscles (Jensen et al., 2017).

### No function related phenotypical differences between trabecular and compact myocardium

Generating contractile force requires a high metabolic rate which has to be supported by the presence of a vast vascular network and a high mitochondrial density. There was no evidence that this was lacking in adult trabecular myocardium as it had similar mitochondrial respiration rates as the compact myocardium and it has long been demonstrated that the papillary myocardium and the trabecular wall are as vascularized as the neighboring compact wall (Estes et al., 1966; Wearn, 1928). From the stance of microscopic anatomy, the initial differences between embryonic trabeculations and embryonic compact myocardium disappear in the older heart in which the trabecular wall also acquires its own vascular network (Sedmera et al., 2000). The absence of an unambiguous marker for trabecular myocardium in the adult heart is consistent with the trabecular and compact myocardium converging on this more similar phenotype (deBoer et al., 2012; Jensen et al., 2017; Moorman et al., 2000; Sizarov et al., 2011). What is more, proteomics and identification of cell types using single cell sequencing identify different trabecular and compact phenotypes and different trabecular and compact cells in embryonic human hearts (Asp et al., 2019; Cui et al., 2019) whereas similar distinctions are not found in adult hearts (Doll et al., 2017; Litviňuková et al., 2020; Tucker et al., 2020). This strongly suggests that trabecular and compact myocardium is largely similar in the adult heart.

### No functional differences between trabecular and compact myocardium

Our results indicate that adult cardiomyocytes from the trabecular and compact layer are equally equipped for force generation which is supported by *in silico*, genetic, and phenotypic correlation assays (Meyer et al., 2020). The maximal generated forces we describe, more than 10 kN/m^2^, correspond to those in other species where they were inferred from systemic blood pressure, wall thickness and ventricular dimensions (Seymour and Blaylock, 2000). Fetal cardiomyocytes were found to be less strong than adult cardiomyocytes, again confirming that adult trabecular myocardium does not have an embryonic identity (Jensen et al., 2017). That the vast majority of people with excessive trabeculations are asymptomatic is consistent with the trabecular and compact myocardium having a similar contractile force potential (Ivanov et al., 2017; Weir-McCall et al., 2016; Zemrak et al., 2014). Modeling of pump function suggests that the presence of trabeculations has a positive impact on the ejected volume (Paun et al., 2018). Also, most vertebrate species have a highly trabeculated ventricle (Anderson et al., 2017). If trabecular and compact myocardium is equally equipped for force generation, how then to understand that excessive trabeculation and non-ischemic cardiomyopathy can co-exist? This could be resolved if the presence of excessive trabeculation and cardiomyopathy are largely or wholly independent of each other. When mutations are recognized in so-called noncompaction, they are frequently found in genes encoding sarcomeric proteins (Mazzarotto et al., 2021; van Waning et al., 2018) and the trabecular and compact myocardium may be equally affected. At least to date, we do not know of studies showing the trabecular layer to be the only one affected by mutations.

Foundational analyses of ventricular structure-function relations omitted the trabeculations because of their high complexity (Streeter, 1979). However, as the discussion above shows, this should not imply that the trabecular layer is irrelevant to function or that its function is inferior to that of the compact layer.

### Limitations of the study

Because of the morphometric analysis sensitivity it is possible that there is still a small contribution of compaction to the growth of the compact wall. Here, lineage tracing could potentially offer more insight if individual compact cardiomyocytes in the older heart can be traced back to the trabecular fraction in the younger heart. Unfortunately, no ubiquitous and exclusive marker of trabecular myocardium in the early embryo is known (Tian et al., 2017). In mouse, the trabecular cardiomyocyte markers *Nppa* and *Gja5*, which have been used previously to demonstrate trabecular wall lineage, only become restricted to the trabecular myocardium around the end of the embryonic period, which is after the moment where we demonstrated the shift in trabecular to compact wall thickness ratio and which would, therefore, be the most interesting period to investigate (Houweling et al., 2005). However, our analysis excludes the possibility of a large contribution of compaction to the growth of the compact wall, and, therefore, it is unlikely that failure of compaction can lead to the extremely trabeculated ventricles observed in some patients with excessive trabeculation (Jenni et al., 2001). Compaction has been previously described for chicken in the right ventricle (Rychterová, 1971) and has been suggested to occur in the left ventricle on the basis of compact wall thickness measurements only (Sedmera et al., 2000). However, we could not replicate the same steep increase in compact wall thickness around day 10 in chick development as reported previously (Sedmera et al., 2000), even when artificially shifting our trabecular to compact borderlines (Online Figure S3). The validity of this founding observation has been questioned (Faber et al., 2021a) and the data we report here suggests a gradual thickening of the compact wall that is not dependent on compaction of trabecular myocardium.

Only a few cases of human excessive trabeculation were studied. These cases appear similar to the type called “spongy” by Burke et al. and different from the types called “anastomosing” and “polypoid” (Burke et al., 2005). Perhaps, then, the cases we have studied are only representative of a fraction of the substantial variation reported under the category of so-called noncompaction.

The three-dimensional configuration of the trabecular and compact myocardium was not taken into account in these measurements. It remains to be investigated if the different vectors of the individual cardiomyocytes in the trabecular wall limit the effect these cells might have on blood-propulsion *in vivo*. In addition, it has not been established what causes the heart failure in those patients with excessive trabeculation that do not have any obvious genetic mutations causing sarcomeric or cytoskeletal protein dysfunction. It is possible that (micro)environmental factors, which might be influenced by cross-talk between endocardium and myocardium, can induce rhythm disorders or heart failure by causing differential gene expression (Costantini et al., 2005). Here, single cell transcriptomic analysis might offer more insight, as has previously been performed for cell-cycle markers in trabecular and compact myocardium in mouse embryo’s (Li et al., 2019).

### Conclusions

We assessed left ventricular trabecular and compact layer growth in human, mouse and chicken. The left ventricular wall acquires its adult proportions of trabecular and compact myocardium by differential growth rather than by compaction (conversion) of trabecular to compact myocardium. Likely, “noncompaction” is a misnomer. We found no difference in trabecular and compact myocardial functional phenotype, in contrast to what is presumed about patients diagnosed with “noncompaction”.

## STAR★Methods

### Key resources table

**Table:** 

REAGENT or RESOURCE	SOURCE	IDENTIFIER
**Antibodies**		

digoxigenin labelled RNA probes against TNNI3 for mouse	Moorman et al., (2001)	N/A
digoxigenin labelled RNA probes against TNNI3 for chicken	Moorman et al., (2001)	N/A
COX4 monoclonal mouse-anti-human	Abcam	20E8C12; RRID:AB_2535839
cardiac troponin I polyclonal goat-anti-human	HyTest	4T21/2; RRID:AB_154084
Donkey-anti-rabbit Alexa 555	Invitrogen	A31572; Lt. 1945911; RRID:AB_162543
Donkey-anti-mouse Alexa 488	Invitrogen	A21202; Lt. 1890861; RRID:AB_141607
CD31 monoclonal mouse-anti-human	Dako	JC70A; RRID:AB_2114471
MAB1273 monoclonal mouse-anti-human	Sigma-Aldrich	RRID:AB_94052

**Biological samples**		

Human hearts	Medical Biology Department archives at Amsterdam UMC.	N/A
Human hearts	University of Extremadura, Spain	N/A
Human hearts	Pathology Department of Amsterdam UMC	N/A

**Chemicals, peptides, and recombinant proteins**		

Triton X-100	Sigma-Aldrich	
Paraffin	Leica	Paraplast
Picric acid saturated 1.3%	Sigma	P6744-1GA
Sirius-red F3B	Gurr	N/A
haematoxylin	Merck	4305
eosin	Merck	1345
Antigen Unmasking Solution	Vector	H3300; Lt. ZB1016
Tris	Invitrogen	N/A
HCl	Merck	N/A
NaCl	Merck	N/A
DAPI	Sigma	D9542
Phosphate buffered saline (PBS)		N/A
Glycerol	Scharlau	GL0027
DAB+	Dako	K3468
Saponin	Miranda-Silva et al., (2020)	N/A
CaEGTA (7.2 mM EGTA, 5.8 mM ATP, 6.6 mM MgCl_2_, 20 mM taurine, 15 mM phosphocreatine, 20 mM imidazole, 0.5 mM dithiothreitol and 50 mM MES buffer (pH 7.1))	Miranda-Silva et al., (2020)	N/A
EGTA (3 mM MgCl_2_, 60 mM K-lactobionate, 20 mM taurine, 10 mM KH_2_PO_4_, 20 mM HEPES, 110 mM sucrose and 1 g/L fatty acid free bovine serum albumin (pH 7.1))	Miranda-Silva et al., (2020)	N/A
ADP	Miranda-Silva et al., (2020)	N/A
cytochrome c	Miranda-Silva et al., (2020)	N/A
succinate	Miranda-Silva et al., (2020)	N/A
FCCP (carbonylcyanide-4-(trifluoromethoxy)-phenylhydrazone)	Miranda-Silva et al., (2020)	N/A
relax solution (1 mM Mg^2+^, KCl 145 mM, 2 mM EGTA, 4 mM ATP, 10 mM imidazole (pH 7.0))	Miranda-Silva et al., (2020)	N/A
Shellac	Van den Velden et al. (2003)	N/A

**Critical commercial assays**		

blocking powder	Perkin & Elmer	TSA Enhancement kit; NEL702A

**Deposited data**		

Raw and analyzed data	This paper	N/A

**Experimental models: Organisms/strains**		

Mouse, wild-type, FVB/NRj	Janvier labs	FVB/NRj
Chicken, wild-type	Drost BV	wild-type

**Software and algorithms**		

ImageJ 1.51j8 software	National Institutes of Health, USA	N/A
GraphPad Prism 8.3.0	GraphPad Software, LLC	N/A

**Other**		

light microscope	Leica	CTR5000
microtome	Leica	N/A
camera	Leica	DFC450
immunofluorescent microscope	Leica	CTR6000
camera	Qimaging	Retiga EXi Fast; SN:Q19688
Confocal microscope	Leica	TCS SP8 SMD
inverted microscope	Leica	DMI6000
objective	Leica	HC PL APO CS2 63×/1.40
respirometer	Oroboros Instruments, Innsbruck, Austria)	Oxygraph-2k
force transducer	Van den Velden et al. (2003)	N/A
piezoelectric motor needle	Van den Velden et al. (2003)	N/A

### Resource availability

#### Lead contact

Further information and requests for resources and reagents should be directed to and will be fulfilled by the lead contact, Bjarke Jensen (b.jensen@amsterdamumc.nl).

#### Materials availability

• This study did not generate new unique reagents.
• This study did not generate new mouse or chicken lines.
• Used reagents are commercially available and the previously in-house generated *in situ* hybridization probes that were used can be acquired by contacting the corresponding author.

### Experimental model and subject details

All studies were in compliance with the Helsinki Declaration and all protocols were approved by the Medical Ethics Committee of the University of Amsterdam, The Netherlands. The study was carried out in compliance with the ARRIVE guidelines and animal care and experiments conformed to the Directive 2010/63/EU of the European Parliament. All animal work was approved by the Animal Experimental Committee of the Amsterdam University Medical Center, location AMC, Amsterdam, and was carried out in compliance with the Dutch government guidelines, approved by the Central Committee Animal Experiments.

#### Hearts

##### Human

For morphometric analysis we used single sections of hearts in approximately 4-chamber view from embryos and fetuses of unknown sex of 3 to 36 weeks of gestation (N = 37). Sections from 20 hearts were derived from already published section series (Sizarov et al., 2010, 2012). Eight sections were derived from the Medical Biology Department archives at Amsterdam UMC. These sections had all been immunofluorescently stained for cardiac troponin I (Sizarov et al., 2010) or histochemically with hematoxylin and eosin. Nine fetal sections, stained with Masson Trichrome, were provided by Dr Sánchez-Quintana, and these hearts were obtained with permission of the Bioethical Committee on Human Research of the University of Extremadura, Spain. The sex of humans from whom these hearts originated is unknown since the tissue had been anonymized after collection. No chromosome analysis has been performed.

Paraffin sections of hearts of aborted fetuses of age 20 to 21 weeks (sex unknown) with either normal (N = 2) or excessive trabeculations in a setting of multiple congenital malformations (N = 3) (Jensen et al., 2017), and sections from adult, autopsy derived, morphologically normal human hearts (N = 6, sex unknown) were obtained from archives of the Pathology Department of Amsterdam UMC, and used for the phenotypical analysis of the myocardium. Informed consent was obtained for all autopsies from the decedent’s next of kin and for the anonymous use of archived autopsy materials a waiver was granted by the Medical Ethics Committee of the University of Amsterdam.

#### Mouse

FVB/NRj wild-type mouse embryos (*Mus musculus*, Janvier labs, www.janvier-labs.com) of 9.5 to 17.5 gestational days were obtained from a variety of in-house bred litters. Hearts from these mice, and from postnatal mice from day 0 to 23 were isolated and fixed with 4% paraformaldehyde in phosphate buffered saline for morphometric analysis (N = 47). Sex of the individuals was unknown.

For the mitochondrial activity assay, fresh samples of left ventricular papillary myocardium with surrounding trabeculations, compact wall, as well as left atria were collected from freshly killed adult surplus female mice (N = 6).

For the contractile force analysis, whole ventricles from 7 fetal mice of unknown sex, at 15.5 gestational days were collected and pooled per litter (N = 2) containing 3 and 4 individuals each to obtain substantial enough tissue volumes. Furthermore, separated left ventricular compact and trabecular myocardium from surplus wild-type adult hearts (N = 6, 3 female, 3 male) was obtained by fine dissection before tissues were being snap frozen in liquid nitrogen.

#### Chicken

Wild-type chicken embryos (*Gallus gallus*, Drost BV, Nieuw-Loosdrecht, The Netherlands) of 2 to 14 gestational days (sex unknown) were obtained from eggs incubated in a humidified rocking stove at 38.5°C (N = 28). We used archived specimens for ages older than 14 days. Whole embryos or isolated hearts were fixed with 4% paraformaldehyde in phosphate buffered saline. In addition, sections of Bouin fixed chicken embryos, previously histochemically stained for troponin bound antibodies (N = 10) or hematoxylin and eosin (N = 6), were included from the Medical Biology Department archives at Amsterdam UMC. Sex of the specimens was unknown.

### Method details

#### Morphometry

##### *In situ* hybridization

After fixation, tissues were embedded in paraffin (Paraplast, Leica) and sectioned at 10 μm thickness with a Leica microtome. Sections at approximately 4-chamber view were then stained with digoxigenin labeled RNA probes (Moorman et al., 2001) against *TNNI3* for mouse (1 ng/μL) and chicken (0.1 ng/μL). Briefly, we used an established protocol (Moorman et al., 2001) where the sections are pre-treated with proteinase K digestion, typically 5–10 min, with longer times for older tissues. The specific hybridization of probe to mRNA is favored by overnight incubation at 70°C, which is enabled by having the probe mix containing 50% formamide. Detection is done by the binding of digoxigenin with primary antibody to which is bound a secondary antibody with conjugated alkaline phosphatase that promotes the formation of blue precipitate from NBT/BCIP. The staining was allowed to proceed as long as non-myocardial tissues, such as valve mesenchyme, remained unstained.

#### Morphometric assessments

If measurements are taken perpendicular to the epicardial layer, the measurement lines do not intersect in one point in the central cavity and this can introduce substantial error in estimating the average radius and thus size of the ventricle and its components. Instead, on images of stained hearts, a wheel with spokes at 30° intervals was projected (Online Figure S1A). The center of the wheel was placed approximately at the center of the left ventricle whereas the spoke of 0° was placed approximately at the atrioventricular canal, the spoke of 180° was located towards the apex of the heart and the spoke of 330° pointed towards the aorta. The thickness of the lumen, the trabeculated myocardial layer, and compact myocardial layer were measured along the spokes of 0° to 210° with ImageJ 1.51j8 software (National Institutes of Health, USA) which allows for later volume calculations, reduces overlapping measurements and reduces interobserver bias (Online Figure S1B). The border between the myocardial layers was determined on sight by following the free lumen to where the crevasses between the trabeculations were deepest.

The surface areas of the compact and trabeculated myocardium between each spoke from 0° to 210° were measured on binary transformed images with the polygon selection tool in ImageJ after the free lumen area had been subtracted by projection of an, as large as possible, ellipse in the ventricular cavity that did not cross any myocardial structures (Online Figure S1). Both the total surface area and the percentage of myocardialized surface area were collected (Online Figure S1D). In case a spoke did not cross any left ventricular myocardium, for example if the 0° spoke lay in the atrioventricular canal, or when parts of the section were damaged, measurements were resumed at the next suitable spoke. In case no clear distinction between blood and myocardium could be made in the histochemically stained sections, no surface area measurements were taken.

To calculate the volume of compact myocardium ($V_{compact}$) and the volume of trabeculations ($V_{trabecular}$) a spherical model was made using the following equations:

(Equation 1) $$V_{compact}=\tilde{\frac{4}{3}\pi r^{3}}\times \tilde{\frac{A_{compact}\times \frac{\%Myocardium_{compact}}{100}}{A_{total}}}$$

(Equation 2) $$V_{trabecular}=\tilde{\frac{4}{3}\pi r^{3}}\times \tilde{\frac{A_{trabecular-lumen}\times \frac{\%Myocardium_{trabecular-lumen}}{100}}{A_{total}}}$$

The tilde stands for median, so the median of each total ventricular volume, $V_{total}=\frac{4}{3}\pi r^{3}$, with each measured total spoke length taken as r, was multiplied with the median of each myocardial fraction of surface area. Because the measured surface areas are not fully myocardial but also contain intertrabecular lumen and coronary artery lumina, the surface areas were corrected for the myocardial occupancy: %Myocardium. For the trabecular fraction, the lumen area, as obtained by placement of the ellipse, had been subtracted.

#### Phenotypical analysis

##### Histochemical staining

Sections (5μm) of formalin fixed paraffin embedded blocks of transmural human adult left ventricular wall were stained for at least one hour with saturated Picrosirius red, with which collagen stains red and muscle orange (the stain was differentiated for 2 min in 0.01 M HCl). Sections were filtered for all colors except red in ImageJ and then converted to binary images. The staining intensity for trabecular and compact layer was then quantified as the grey scale value of the area trabecular and compact area respectively.

#### Immunofluorescence and immunohistochemistry

Sections of human fetal and adult left ventricles were rehydrated as described for *in situ* hybridization. After rinsing, the samples were cooked in unmasking solution (citrate based Antigen Unmasking Solution, Vector, H3300, Lt. ZB1016). Samples were either incubated with Triton 0.5% and then TNT (0.1M Tris-HCl, pH 7.5; 0.15M NaCl; 0.05% Tween) or immediately in TNT if a membrane antibody was used. Hereafter, samples were blocked with TNB (0.1M Tris-HCl, pH 7.5; 0.15M NaCl; 0.5% blocking powder (TSA Enhancement kit, Perkin & Elmer NEL702A)) for at least 30 minutes. Sections were then incubated with primary antibodies against mitochondrial complex IV subunit of the mitochondrial respiratory chain COX4 (1:200, monoclonal mouse-anti-human, Abcam, 20E8C12, RRID:AB_2535839), and cardiac troponin I cTnI (1:200, polyclonal goat-anti-human, HyTest, 4T21/2, RRID:AB_154084) at room temperature overnight. The next day, sections were rinsed in TNT and incubated in the dark with secondary antibodies Donkey-anti-rabbit Alexa 555 (1:250, 2mg/mL 09-08-18, Invitrogen A31572, Lt. 1945911, RRID:AB_162543) and Donkey-anti-mouse Alexa 488 (1:250, 2mg/mL 13-10-17, Invitrogen A21202, Lt. 1890861, RRID:AB_141607). After 2 h they were washed again 3× for 5 min in TNT 1× before being mounted with DAPI (1:250) in PBS/glycerol (1:1). For detection of endothelium and mitochondrial-surface protein we used a primary antibody against CD31 (clone JC70A, Dakopatts, Denmark, RRID:AB_2114471) and MAB1273 respectively (1:100, monoclonal mouse-anti-human, Sigma-Aldrich, RRID:AB_94052), which were visualized with DAB+ (Dako) in an immunoperoxidase stain and faintly counterstained with hematoxylin.

Images of sections were imported to ImageJ and converted to 8 bit (grey scale) images. In the adult human samples, staining intensities in the trabecular layer, compact layer and epicardium were calculated respectively as their average grey scale values, which informed us on the relative amount of detected protein in each different tissue area per section. For the human fetal specimens, the signal intensity of five (epicardium) to ten (trabecular and compact) areas within a single section were corrected to the median intensity of the 25 measurements per section. First, we measured within the cross sections of ten trabeculations that were widely spaced from each other. Secondly, we measured the compact and epicardial tissues using areas that were approximately the same in size as those measured in the trabeculations. For the compact wall and epicardium, we chose areas for measurements that were widely distributed within each section. The shown comparisons are the corrected average values of five (epicardium) to ten (trabecular and compact) areas per section.

#### Functional analysis

##### Mitochondrial respiration

From 6 adult mice, left ventricular papillary myocardium with surrounding trabecular wall, compact wall, and left atrium were isolated. Mitochondrial respiration in these tissues was assessed using an established protocol (Miranda-Silva et al., 2020). Briefly, samples were permeabilized with 50 μg/mL saponin for 30 min at 4°C in a preservation solution consisting of 2.8 mM CaEGTA, 7.2 mM EGTA, 5.8 mM ATP, 6.6 mM MgCl_2_, 20 mM taurine, 15 mM phosphocreatine, 20 mM imidazole, 0.5 mM dithiothreitol and 50 mM MES buffer (pH 7.1). Tissue was subsequently washed in respiration solution, containing 0.4 mM EGTA, 3 mM MgCl_2_, 60 mM K-lactobionate, 20 mM taurine, 10 mM KH_2_PO_4_, 20 mM HEPES, 110 mM sucrose and 1 g/L fatty acid free bovine serum albumin (pH 7.1), quickly blotted dry, weighed and transferred to a respirometer (Oxygraph-2k, Oroboros Instruments, Innsbruck, Austria) in respiration solution at 37°C. Oxygen concentration was maintained above 300 μM throughout the experiment to avoid limitations in oxygen supply. Leak respiration, determined as respiration with substrates but without ADP, was assessed after addition of 10 mM sodium glutamate, 0.5 mM sodium malate and 5 mM sodium pyruvate. Maximal NADH-linked (via complex I) respiration was assessed after the addition of 2.5 mM ADP and cytochrome c. Outer-mitochondrial membrane damage was absent as no sample showed an increase of >15% in respiration after the addition of 10 μM cytochrome c. Maximal oxidative phosphorylation (OXPHOS) capacity was measured after addition of 10 mM succinate. Maximal uncoupled respiration was measured after the stepwise addition of 0.01 μM FCCP (carbonylcyanide-4-(trifluoromethoxy)-phenylhydrazone). Respiration values were normalized to wet weight and expressed in pmol O_2_·s^−1^·mg^−1^.

#### Force generation

To investigate if trabecular and compact myocardium could be functionally different, mouse heart samples were subjected to calcium induced contraction force measurements from which maximal active (F_max_) and passive force (F_pas_) as well as the Hill coefficient (nHill), and EC_50_, were obtained.

The snap-frozen samples of mouse ventricles, from six adult hearts and two fetal hearts, were defrosted and ground up in relax solution containing 1 mM Mg^2+^, KCl 145 mM, 2 mM EGTA, 4 mM ATP, 10 mM imidazole (pH 7.0). After a 5 min incubation in 0.5% triton X-100 in relax solution, single adult cardiomyocytes or small clusters of fetal cardiomyocytes were selected on the basis of a minimal width of 20μm and a maximal width of 61μm, with visible sarcomeric structures to be measured after a force transducer and a piezoelectric motor needle were glued onto the cell with shellac.

Calcium response curves were measured at a sarcomeric length of 2.2 μm (Van der Velden et al., 2003). To this end, glued cells or cell clusters were transferred into a series of solutions with a [Ca^2+^] of 10^−4.5^, 10^−4.5^, 10^−5.4^, 10^−6^, 10^−5.2^, 10^−5.8^, 10^−5^, 10^−6.2^, and 10^−4.5^ M respectively. The total force was the combined active (F_act_) and passive force (F_pas_) with F_pas_ being the force generated after transferring the tissue back to relax solution and slackening the tissue length by 30%. The maximal force (F_max_) was the F_act_ obtained during the second subjection to [Ca^2+^] 10^−4.5^ M. All forces were corrected for cell cross-sectional area (A=width×depth×π). The Hill coefficient (nHill), and EC_50_, were calculated from the established relative calcium response curves in GraphPad prism using a non-linear curve fit for the dose-response curve agonist versus response – variable slope (four parameters) using 1 and 0 as top and bottom constraints. If the generated force at the last [Ca^2+^] of 10^−4.5^ M was reduced by more than 30% compared to the second [Ca^2+^] of 10^−4.5^ M, we deemed the preparation was deteriorated and it was not included in the subsequent analyses.

#### Imaging

A Leica CTR5000 light microscope with a DFC450 camera was used to image the histochemical and *in situ* hybridization stains. Immunofluorescent stains were imaged using a Leica CTR6000 microscope mounted with a Retiga EXi Fast camera (SN:Q19688, QImaging). Confocal microscopy was performed with a Leica TCS SP8 SMD mounted on a Leica DMI6000 inverted microscope with objective HC PL APO CS2 63×/1.40.

### Quantification and statistical analysis

Analyses were performed using GraphPad Prism 8.3.0 (GraphPad Software, LLC). Error bars for the thickness slopes represent 95% confidence intervals. In all other figures error bars represent standard deviations and lines represent means. Growth curves were compared with linear regression analyses and correlation of thickness growth with age was calculated with a Pearson correlation coefficient. Staining signal intensities were compared with Tukey corrected One-Way ANOVA. Functional outputs were compared with Tukey’s corrected one-way ANOVA or mixed-effects analysis. Further details on statistics used can be found in the figure legends, in the experimental model and subject details and in the method details. Values of p < 0.05 were considered significant, ∗ = p ≤ 0.05, ∗∗ = p ≤ 0.01, ∗∗∗ = p ≤ 0.001.

## Data Availability

• All data reported in this article will be shared by the lead contact on request. • This article does not report original code. Any additional information required to reanalyze the data reported in this article is available from the lead contact on request.
