# Supplementary material for: Equal force generation potential of trabecular and compact wall ventricular cardiomyocytes
Source: iScience. 2022 Oct 17;25(11):105393. doi: 10.1016/j.isci.2022.105393 (PMC9636041; doi:10.1016/j.isci.2022.105393)
Supplement: Document S1. Figures S1–S3 [file mmc1.pdf]

iScience, Volume *25*

Supplemental information

Equal force generation potential of trabecular and compact wall ventricular cardiomyocytes

Jaeike W. Faber, Rob C.I. Wüst, Inge Dierx, Janneke A. Hummelink, Diederik W.D. Kuster, Edgar Nollet, Antoon F.M. Moorman, Damián Sánchez-Quintana, Allard C. van der Wal, Vincent M. Christoffels, and Bjarke Jensen

Supplemental items

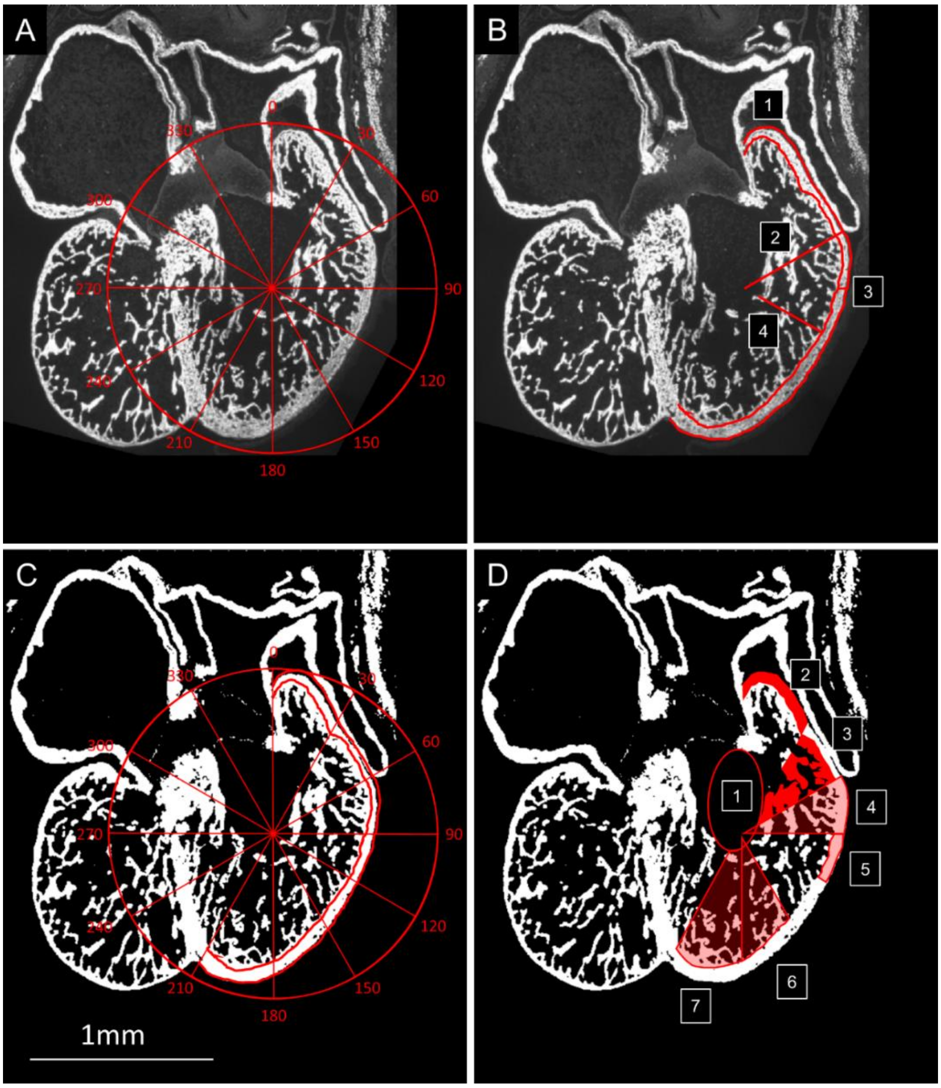

Online Figure 1. 2D measurements, related to STAR methods. A. Immunofluorescent stained section of a 45 day old human embryo with a superimposed measuring wheel with spokes at 30° intervals. **B**. 1: outer margin of the free wall of the left ventricle and borderline between compact and trabecular myocardium; 2: total spoke length along 60° spoke; 3: compact thickness along 90° spoke; 4: trabecular thickness along 120° spoke. **C**. Binary transformed section with the superimposed measuring wheel. **D**. 1: ellipse demarcating lumen area; 2: compact myocardial area (red) between 0° and 30°; 3: trabecular myocardial area (red) between 30° and 60°; 4: total ventricular area (shaded red) between 60° and 90°; 5: compact area (shaded red) between 90° and 120°; 6: trabecular plus lumen area (shaded red) between 150° and 180°; 7: trabecular minus lumen area between 180° and 210°. All shown images are on the same scale (scale bar 1 mm).

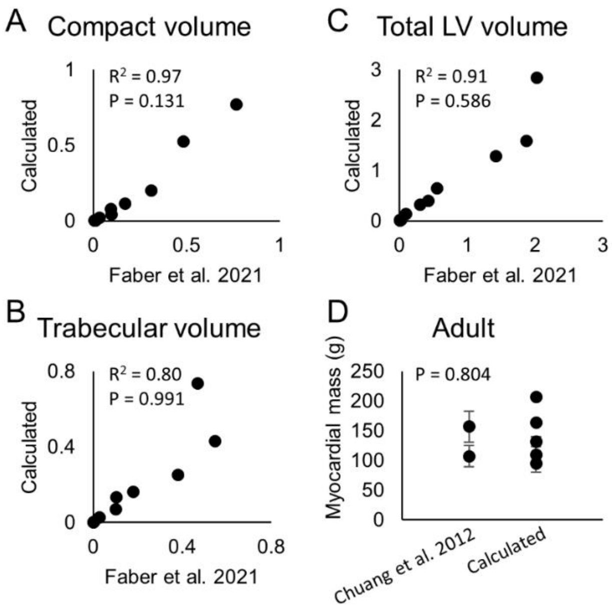

Online Figure 2. Calculated volumes correlated with previously published volumes, correlation with Figure 1. **A**-**C**. Our calculated compact left ventricular volumes (A), trabecular left ventricular volumes (B), and total left ventricular volumes, including lumen (C) were highly correlated and not significantly different (two-tailed unpaired Student's T-tests, N=10), to the corresponding absolute volumes of the same specimens as previously described (Faber et al., 2021b). **D**. Previously provided values on total left ventricular mass (Chuang et al., 2012) in adults were not significantly different from our calculated total left ventricular myocardial masses in adults when a myocardial density of 1.05 g/ml was applied (Grothoff et al., 2012; Papavassiliu et al., 2005) (p=0.792, two-tailed unpaired Student's T-test, data represented as mean ± standard deviation).

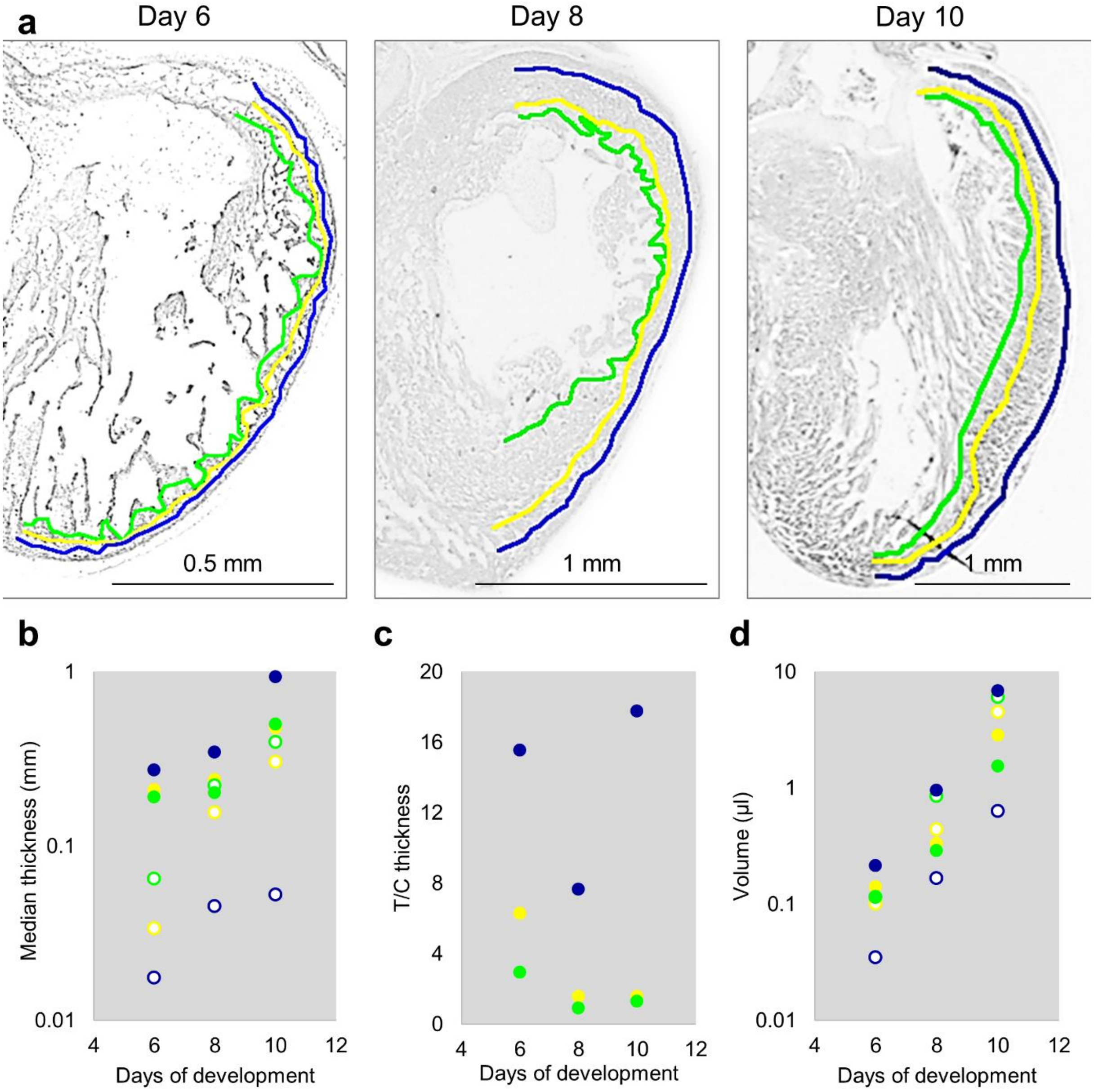

Online Figure 3. Effect of artificial border change to growth curves in chicken, correlation with Figure 2. When the best-fit border (yellow) between trabecular and compact wall was artificially moved (A) to augment the trabecular layer with compact (blue) or to augment the compact layer with trabeculations (green), the effect on trabecular (open circle) and compact wall (closed circle) layer thickness (B), ratio (C), and volume (D) changed accordingly. If the analysis is skewed towards detecting compaction, by measuring an augmented trabecular layer at day 6 (blue) and an augmented compact layer at day 10 (green), the trabecular volume at day 6 was still smaller than at day 10 and no compaction was detected. This indicates that the overall ventricular growth obscures any subtle process of possible compaction. That ventricular growth is substantial between days 6 and 10 is also captured in the difference in scale of the images shown in A, notice the differences in the scale bars.
